# Defining Comprehensive Disease Control for Use as a Treatment Target for Ulcerative Colitis in Clinical Practice: International Delphi Consensus Recommendations

**DOI:** 10.1093/ecco-jcc/jjad130

**Published:** 2023-08-16

**Authors:** Stefan Schreiber, Silvio Danese, Axel Dignass, Eugeni Domènech, Massimo C Fantini, Marc Ferrante, Jonas Halfvarson, Ailsa Hart, Fernando Magro, Charlie W Lees, Salvo Leone, Marieke J Pierik, Michele Peters, Polly Field, Helen Fishpool, Laurent Peyrin-Biroulet

**Affiliations:** University Hospital Schleswig-Holstein, Department of Internal Medicine I, Kiel, Germany; Gastroenterology and Endoscopy, IRCCS Ospedale San Raffaele and Vita-Salute San Raffaele University, Milan, Italy; Department of Medicine I, Agaplesion Markus Hospital, Goethe University, Frankfurt, Germany; Department of Gastroenterology, Hospital Universitari Germans Trias i Pujol and CIBEREHD, Badalona, Spain; Departament de Medicina, Universitat Autònoma de Barcelona, Barcelona, Spain; Department of Medical Science and Public Health, University of Cagliari, Cagliari, Italy; Department of Gastroenterology and Hepatology, University Hospitals Leuven, KU Leuven, Leuven, Belgium; Department of Gastroenterology, Faculty of Medicine and Health, Örebro University, Örebro, Sweden; IBD Unit, St. Mark’s Hospital, London, UK; CINTESIS@RISE, Faculty of Medicine of the University of Porto, Porto, Portugal; Edinburgh Inflammatory Bowel Disease Unit, Western General Hospital, Edinburgh, UK; European Federation of Crohn’s & Ulcerative Colitis Associations [EFCCA], Brussels, Belgium; Division Gastroenterology and Hepatology, Maastricht University Medical Centre, Maastricht, The Netherlands; Nuffield Department of Population Health, University of Oxford, Oxford, UK; Oxford PharmaGenesis, Oxford, UK; Oxford PharmaGenesis, Oxford, UK; Department of Gastroenterology, Nancy University Hospital, Vandœuvre-lès-Nancy, France; Inserm, NGERE, University of Lorraine, Nancy, France; INFINY Institute, Nancy University Hospital, Vandœuvre-lès-Nancy, France; FHU-CURE, Nancy University Hospital, Vandœuvre-lès-Nancy, France; Groupe Hospitalier privé Ambroise Paré – Hartmann, Paris IBD Center, Neuilly sur Seine, France; Division of Gastroenterology and Hepatology, McGill University Health Centre, Montreal, QC, Canada

**Keywords:** Ulcerative colitis, remission, Delphi consensus, patient-reported symptoms

## Abstract

**Background and Aims:**

Treatment of ulcerative colitis [UC] requires a patient-centric definition of comprehensive disease control that considers improvements in aspects not typically captured by classical landmark trial endpoints. In an international initiative, we reviewed aspects of UC that affect patients and/or indicate mucosal inflammation, to achieve consensus on which aspects to combine in a definition of comprehensive disease control, using a modified Delphi process.

**Methods:**

The Delphi panel comprised 12 gastroenterologists and one patient advocate. Two gastroenterologists were elected as chairs and did not vote. To inform statements, we asked 18 patients and the panel members about their experiences of remission and reviewed published literature. Panel members voted on statements anonymously in three rounds, with a live discussion before Round 3. Consensus was met if ≥67% of the panel agreed. Statements without consensus in Rounds 1 and 2 were revised or discarded after Round 3.

**Results:**

The panel agreed to measure individual patient benefit using a definition of comprehensive disease control that combines aspects currently measured in trials [rectal bleeding, stool frequency, disease-related quality of life, endoscopy, histological inflammatory activity, inflammatory biomarkers, and corticosteroid use] with additional patient-reported symptoms [bowel urgency, abdominal pain, extraintestinal manifestations, fatigue, and sleep disturbance]. The panel agreed on scoring systems and thresholds for many aspects.

**Conclusions:**

Using a robust methodology, we defined comprehensive disease control in UC. Next, we will combine the measurement and scoring of these aspects into a multicomponent tool and will adopt comprehensive disease control as a treatment target in clinical practice and trials.

## Graphical Abstract

**Graphical Abstract:**
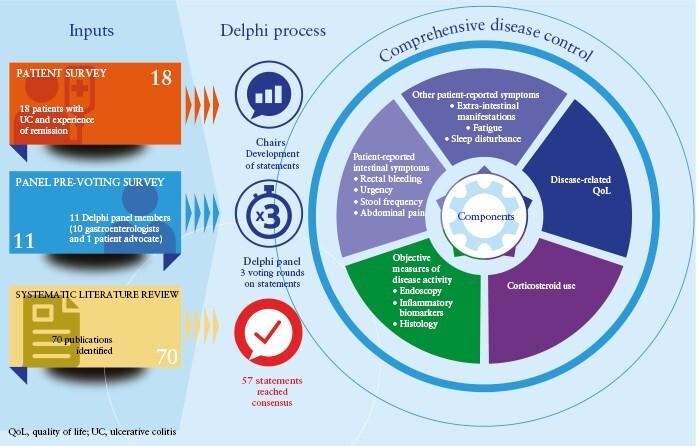

## 1. Introduction

Ulcerative colitis [UC] can have a major impact on patients’ lives even when they are in clinical remission, solely as defined by standard criteria based on the Mayo score.^1^ Residual gastrointestinal symptoms, such as urgency, as well as non-gastrointestinal symptoms, such as fatigue, mental exhaustion, anxiety, depression, and sleep disturbance,^2–5^ may still affect how patients live their lives, their ability to work and interact socially, and their general health.^6^ However, these symptoms are accorded less importance because clinical practice guidelines^7,8^ have adopted the academic and regulatory definition of symptomatic remission, which in clinical practice is based on stool frequency and rectal bleeding.^9^

The Selecting Therapeutic Targets in Inflammatory Bowel Disease [STRIDE] II initiative^9^ recommended: evaluating stool frequency and rectal bleeding as short-term targets; normalisation of inflammatory biomarkers (faecal calprotectin [FC] and C-reactive protein [CRP]) as intermediate treatment targets; and normalisation of quality of life [QoL] and endoscopic remission as long-term treatment targets.^9^ Histological and transmural healing were not recommended as treatment targets, but histological healing was instead recommended as an adjunct to endoscopic remission to represent a deeper level of healing.^9^ These targets align with regulatory advice for assessing treatment efficacy, based on achieving and maintaining clinical remission.^10^ However, they do not include many of the physical and psychological aspects of UC that affect patients.

We suggest that treatment of UC requires a patient-centric approach and a holistic definition of ‘comprehensive disease control’ that goes beyond standard regulatory definitions of clinical remission. This should capture aspects of UC that are important to patients and should consider other measures that are predictive of long-term outcomes, such as endoscopic findings or inflammatory biomarkers.

We followed a modified Delphi process to build on the recommendations from STRIDE II^9^ and reach consensus on the aspects of UC that could be combined in a definition of ‘comprehensive disease control’ and used as a treatment target; we also provide guidance on specific measurement tools and thresholds.^9,11^

## 2. Methods

### 2.1. Pre-Delphi research

#### 2.1.1. Patient survey

We captured patient opinions using an online survey and supplemented this with a review of published research into patient preferences. Participants were required to complete a consent form and confirm that they were aged 18 years or older, had UC, had experience of remission, and could understand English before being able to view the questionnaire. The survey was open between 21 April and 18 May 2022. Additional information on the patient survey can be found in the Supplementary materials. In total, 18 patients answered questions about the symptoms they experienced while in remission, how these affected their daily lives, and what they perceived remission to mean. Responses to the survey were aggregated, and all data were anonymised. Patient characteristics can be found in Supplementary Table 6.

#### 2.1.2. Systematic and targeted reviews

A systematic literature review [SLR] identified evidence for adult patients with UC in clinical remission. Information on the methodology can be found in the Supplementary materials. In total, 70 papers met the inclusion criteria, as shown in the PRISMA diagram in Supplementary Figure 1. Studies not captured by the SLR were identified from the STRIDE II publication^9^ and targeted literature reviews [TLRs].

### 2.2. Delphi process

#### 2.2.1. Delphi panel

We followed a modified Delphi consensus process to establish consensus.^12,13^ The Delphi consensus panel comprised 12 gastroenterologists who were identified and invited to participate based upon their expertise, participation in recent clinical trials, and willingness to contribute. The panel also included one patient advocate who had experience of UC in remission. Two of the gastroenterologists on the Delphi panel, Professor Stefan Schreiber and Professor Laurent Peyrin-Biroulet, were elected to chair the entire process and oversaw all research activities and development of the statements, but they did not participate in the voting. An expert on patient-reported outcomes [PROs], Dr Michele Peters, provided guidance on the inclusion of patient-reported outcome measures [PROMs] in the statements but did not participate in the voting.

#### 2.2.2. Pre-voting survey

Delphi panel members [excluding the two chairs] completed a survey about UC symptoms during remission, based on clinical or personal experience. Gastroenterologists on the panel also answered questions about evaluating disease activity using clinical measures, such as endoscopy and inflammatory biomarkers.

#### 2.2.3. Statement development

A summary of the evidence that supported the statements from the symptom survey, the SLR, the TLRs, and the Delphi panel pre-voting survey were all available to all Delphi panel members in the form of a briefing book. This evidence informed ‘statements’ that were developed by the chairs and voted on by the other Delphi panel members. Note, some statements asked about measurement tools for symptoms, and the selection of tools was informed by practical considerations about completing the tools. Thus, when developing the statements, the chairs informally assessed the feasibility of completing assessments for each symptom, considering that each patient may have many symptoms to assess.

#### 2.2.4. Voting rounds

The Delphi comprised three rounds of virtual voting. Responses were given on a six-point scale [strongly disagree, disagree, somewhat disagree, somewhat agree, agree, or strongly agree] in voting Rounds 1 and 2. Statements that met consensus, either to agree [achieved a response of strongly agree, agree, or somewhat agree], disagree [strongly disagree, disagree, or somewhat disagree], or had a yes or no response by ≥67% of the panel in a voting round were included in the final list of statements and were not included in the next voting round. Statements that did not meet consensus were amended based on feedback from the Delphi consensus panel and were included in the next round of voting. Before the third and final voting round, a live discussion took place at a virtual meeting, during which the statements were amended and participants voted to agree or disagree with the amended statements. After the third voting round, statements that did not meet consensus were rejected.

## 3. Results

**Figure 1.**
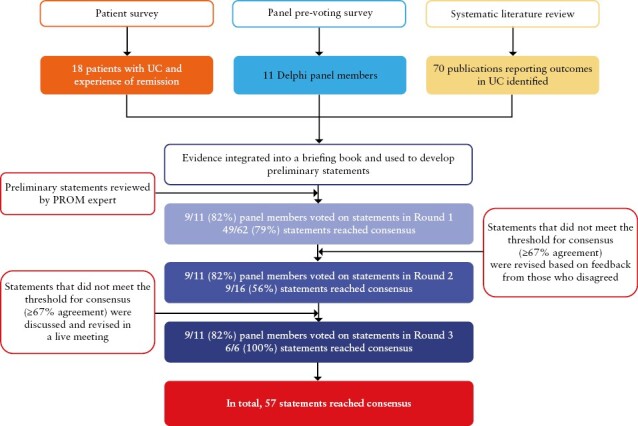
Flowchart of Delphi process. PROM, patient-reported outcome measure; UC, ulcerative colitis.

In total, nine panel members voted in each of the three voting rounds. At the end of the process, the Delphi panel had reached consensus on 57 statements [Figure 1]. A full summary of the changes made to statements between voting rounds can be found in Supplementary Table 7.

### 3.1. PROs

**Table:** 

Consensus statements
**Rectal bleeding**
**Rectal bleeding is important to consider when assessing comprehensive disease control** Consensus: Round 1, 9/9 agreed [100%]; strength^a^ 5.9 [6]
**Assessment of rectal bleeding should be included in a measure of comprehensive disease control** Consensus: Round 1, 9/9 agreed [100%]; strength^a^ 6.0 [6]
**The rectal bleeding component of the two-item, patient-reported outcome [PRO2]/partial Mayo Clinic Score [pMCS] can be used when assessing the severity and frequency of rectal bleeding** Consensus: Round 1, 8/9 agreed [88.9%]; strength^a^ 5.0 [5–6]
**The threshold for comprehensive disease control should be no rectal bleeding, PRO2/pMCS rectal bleeding domain score 0** Consensus: Round 1, 9/9 agreed [100%]; strength^a^ 5.7 [5–6]
**Urgency**
**Urgency is important to consider when assessing comprehensive disease control** Consensus: Round 1, 9/9 agreed [100%]; strength^a^ 5.6 [5–6]
**Urgency should be included in a measure of comprehensive disease control** Consensus: Round 1, 9/9 agreed [100%]; strength^a^ 5.4 [5–6]
**A numerical scale or scoring system [eg, using the Urgency numerical rating scale measurement tool] can be used when assessing the severity and frequency of urgency** Consensus: Round 1, 7/9 agreed [77.8%]; strength^a^ 4.6 [4–5]
**Urgency should be absent for remission to be considered comprehensive, with the exception of mild urgency, if patients do not find this impactful** Consensus: Round 3, 9/9 agreed [100%]; strength^a^ N/A^b^
**Stool frequency/diarrhoea**
**Stool frequency/diarrhoea is important to consider when assessing comprehensive disease control** Consensus: Round 1, 9/9 agreed [100%]; strength^a^ 5.7 [5–6]
**Assessment of stool frequency/diarrhoea should be included in a measure of comprehensive disease control** Consensus: Round 1, 9/9 agreed [100%]; strength^a^ 5.8 [6]
**Stool frequency/diarrhoea can be assessed from counting the number of stools per day** Consensus: Round 1, 9/9 agreed [100%]; strength^a^ 5.4 [5–6]
**Stool frequency/diarrhoea can be assessed from counting the number of stools per day and using the thresholds in PRO2/pMCS** Consensus: Round 1, 8/9 agreed [88.9%]; strength^a^ 4.6 [4–5]
**The threshold signifying remission should be 1–2 stools per day more than is normal for them or PRO2/pMCS stool frequency domain score 1** Consensus: Round 1, 7/9 agreed [77.8%]; strength^a^ 4.4 [4–5]
**Abdominal pain**
**Abdominal pain is important to consider when assessing comprehensive disease control** Consensus: Round 1, 8/9 agreed [88.9%]; strength^a^ 4.7 [4–5]
**Abdominal pain should be included in a measure of comprehensive disease control** Consensus: Round 1, 7/9 agreed [77.8%]; strength^a^ 4.2 [4–5]
**A simple system can be used when assessing the severity and frequency of abdominal pain [eg, none, mild, moderate, or severe; 0 to 10]** Consensus: Round 1, 9/9 agreed [100%]; strength^a^ 4.4 [4–5]
**A numerical scale [eg, using the visual analogue scale] can be used when assessing the severity and frequency of abdominal pain** Consensus: Round 1, 8/9 agreed [88.9%]; strength^a^ 4.4 [4–5]
**Abdominal pain should be absent for remission to be considered comprehensive, with the exception of mild abdominal pain, if patients do not find this impactful** Consensus: Round 3, 9/9 agreed [100%]; strength^a^ N/A^b^
**Disease-related QoL**
**Disease-related QoL is important to consider when assessing comprehensive disease control** Consensus: Round 1, 9/9 agreed [100%]; strength^a^ 5.6 [5–6]
**Disease-related QoL should be included in a measure of comprehensive disease control** Consensus: Round 1, 9/9 agreed [100%]; strength^a^ 5.4 [5–6]
**The SIBDQ or IBD-Disk should be used to measure disease-related QoL** Consensus: Round 1, 7/9 agreed [77.8%]; strength^a^ 4.2 [4–5]
**The threshold for comprehensive disease control for disease-related QoL should be no disability, or IBD-Disk ≤24** Consensus: Round 1, 7/9 agreed [77.8%]; strength^a^ 4.2 [4–5]
**Disease-related QoL [eg, assessed using the Short Inflammatory Bowel Disease Questionnaire [SIBDQ] or IBD-Disk] is more important than health-related QoL [eg, assessed using the EQ-5D or SF-36]** Consensus: Round 2, 8/9 agreed [88.9%]; strength^a^ 5.0 [5–6]
**Extraintestinal manifestations**
**Extraintestinal manifestations are important to consider when assessing comprehensive disease control** Consensus: Round 1, 9/9 agreed [100%]; strength^a^ 5.3 [5–6]
**Extraintestinal manifestations should be included in a measure of comprehensive disease control** Consensus: Round 1, 8/9 agreed [88.9%] strength^a^ 5.1 [5–6]
**Fatigue**
**Fatigue is important to consider when assessing comprehensive disease control** Consensus: Round 1, 7/9 agreed [77.8%]; strength^a^ 4.6 [4–5]
**Fatigue should be included in a measure of comprehensive disease control** Consensus: Round 1, 8/9 agreed [88.9%]; strength^a^ 4.4 [4–5]
**A simple system can be used when assessing the severity and frequency of fatigue [eg, none, mild, moderate, or severe; 0 to 10]** Consensus: Round 1, 8/9 agreed [88.9%]; strength^a^ 4.2 [4]
**A numerical scale/scoring system (eg, the IBD-fatigue [IBD-F]/IBD-F SCORE 1 component [evaluated level and duration of fatigue] or Functional Assessment of Chronic Illness Therapy—Fatigue [FACIT-F]] can be used when assessing the severity and frequency of fatigue** Consensus: Round 1, 7/9 agreed [77.8%]; strength^a^ 4.2 [4–5]
**For patients who experienced impactful fatigue when their UC was active, comprehensive disease control should be characterised by a meaningful reduction in fatigue, excluding any fatigue resulting from other obvious non-UC-related causes** Consensus: Round 3, 9/9 agreed [100%]; strength^a^ N/A^b^
**Sleep disturbance**
**Sleep disturbance is important to consider when assessing comprehensive disease control** Consensus: Round 2, 7/9 agreed [77.8%]; strength^a^ 3.8 [4]
**Sleep disturbance should be included in a measure of comprehensive disease control** Consensus: Round 3, 9/9 agreed [100%]; strength^a^ N/A^b^
**If evaluating sleep disturbance, a numerical scale/scoring system [eg, using an instrument like the Patient-Reported Outcomes Measurement Information System [PROMIS] Sleep Disturbance Item Bank** **^39^** **] can be used** Consensus: Round 2, 9/9 agreed [100%]; strength^a^ 4.7 [4–5]
**For patients who experienced impactful sleep disturbance when their UC was active, comprehensive disease control should be characterised by a meaningful reduction in sleep disturbance, excluding any sleep disturbance resulting from other obvious non-UC-related causes** Consensus: Round 3, 9/9 agreed [100%]; strength^a^ N/A^b^

a Strength of recommendation: mean, and interquartile range [IQR] of six-point Likert scale response.

b Responses were not provided on a six-point Likert scale in voting Round 3.

The patient and pre-voting surveys, and literature reviews informed which symptoms were included in the Delphi statements [Table 1].

**Table 1.** Rationale for including PROs in statements [ranked by order of importance, as voted on by panel].

Aspect	Results from patient survey	Results from literature review
**Rectal bleeding**		
** Frequency during remission**	22%	–
** % of patients who rated this symptom as important**	57%	• 26%^14,a^ • 7.1/10 [higher scores indicate that the symptom is more bothersome]^40^
**Urgency**		
** Frequency during remission**	56%	–
** % of patients who rated this symptom as important**	73%^b^	72%^14,a^
**Stool frequency/diarrhoea**		
** Frequency during remission**	44%	56%^5^
** % of patients who rated this symptom as important**	70%^b^	51%^14,a^
**Abdominal pain**		
** Frequency during remission**	39%	51%^5^
** % of patients who rated this symptom as important**	27%^b^	56%^14,a^
**Disease-related QoL**		
	–	• Gastrointestinal symptoms during remission were associated with: ◦ depressive symptoms [*p* <0.001] ◦ anxiety [*p* <0.001] ◦ fatigue [*p* <0.001] ◦ sleep disturbances [*p* <0.001] ◦ main contributor to poor physical and social wellbeing^5^ • Patients with UC in remission with symptoms have worse QoL and are more likely to experience depression or anxiety than those who are asymptomatic^41^
**Extraintestinal manifestations**		
** Frequency during remission**	• Skin symptoms: 22% • Joint symptoms: 39% • Eye symptoms: 17%	–
** % of patients who rated this symptom as important**	• Skin symptoms: 50% • Joint symptoms: 57% • Eye symptoms: 40%	–
**Fatigue**		
** Frequency during remission**	72%	–
** % of patients who rated this symptom as important**	40%^b^	59%^14,a^
**Sleep disturbance**		
** Frequency during remission**	22%	–
** % of patients who rated this symptom as important**	33%^b^	–

Please note, this represents the key supporting evidence; the impact of symptoms on patients was not searched for systematically.

QoL, quality of life; PRO, patient-reported outcome; UC, ulcerative colitis.

^a^In Rubin *et al*.,^14^ patients with UC [not necessarily in remission] were asked which symptoms had a large impact on their QoL.

^b^The patient survey evaluated 18 patients in remission, and patients with the symptom were asked if that symptom was important or very important to them.

#### 3.1.1. Pre-Delphi research—patient and pre-voting surveys

**Figure 2.**
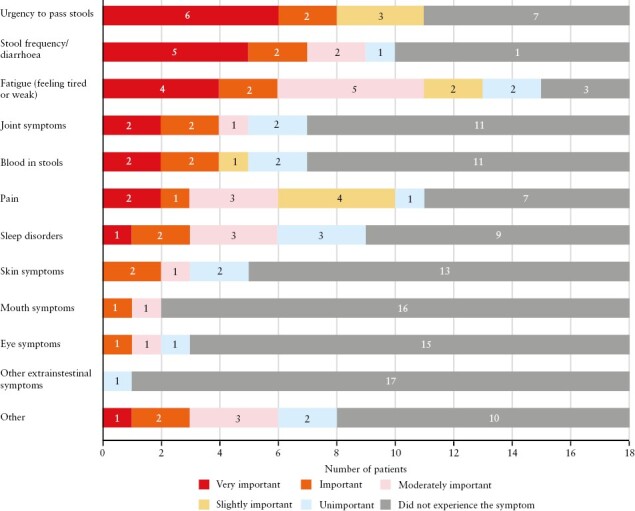
Symptoms experienced by patients with UC who are in remission, and their importance in the patient survey [*N* = 18]. Question: If you have experienced these symptoms when your doctor has told you that you are in remission, how important was each symptom to you? This includes how the symptom made you feel and if it affected your daily activities, such as working or socialising. UC, ulcerative colitis.

Among the symptoms evaluated in our patient survey, urgency was voted to be very important by the most patients [55% of the 11 patients who experienced the symptom; Figure 2], followed by stool frequency/diarrhoea [50% of the 10 patients who experienced the symptom], rectal bleeding and joint symptoms [for each symptom, 29% of the seven patients who experienced the symptom], and fatigue [27% of the 15 patients who experienced the symptom]. Fatigue was the symptom experienced by the most patients [83%].

**Figure 3.**
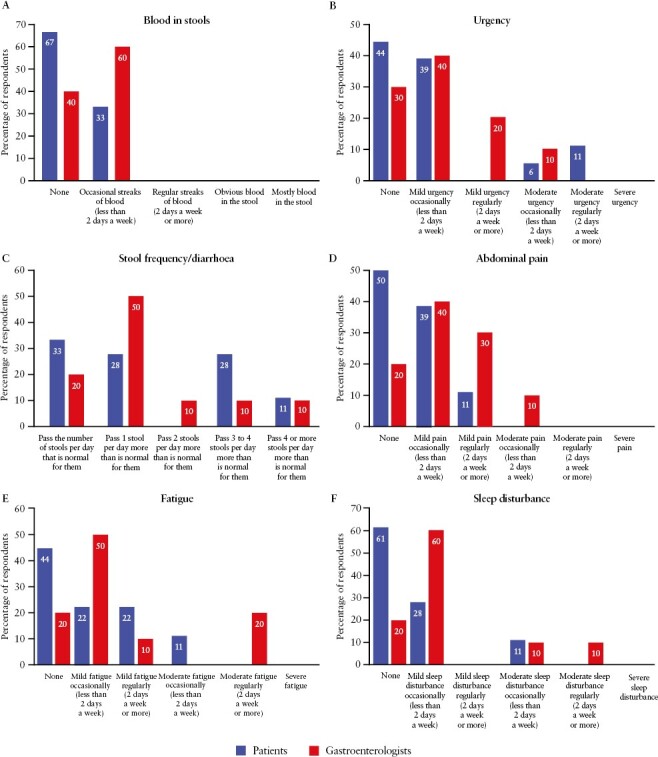
Symptoms threshold for the patient with UC or physician to consider themselves/the patient to be in remission. Question to patients: Please suggest the [level of symptom] that you could experience to consider yourself free from symptoms that affect how you feel and how you live your life. Question to Delphi panel: Please indicate the highest [level of symptom] that your patients could experience and you would still consider them to be in remission. UC, ulcerative colitis.

In our patient survey and pre-voting survey, patients and gastroenterologists were asked for the lowest symptom level that they or their patients could experience while considering themselves or their patients, respectively, to be in remission. In the responses, we saw discrepancies between patients and gastroenterologists [Figure 3]: patients most commonly voted that they would accept no rectal bleeding, no urgency, no increased stool frequency/diarrhoea, no abdominal pain, no fatigue, and no sleep disturbance to consider themselves to be in remission. In contrast, gastroenterologists most commonly voted that they would accept streaks of blood in the stool, mild urgency, mild abdominal pain, mild fatigue, mild sleep disturbance, and passing one stool per day more than is normal, if these symptoms occurred occasionally [less than 2 days a week].

#### 3.1.2. Pre-Delphi research—literature review

Our findings from the patient survey align with the literature [Table 1], with a published survey of 1030 patients with UC finding that rectal urgency [72%], fatigue [59%], abdominal pain [56%], and bloody diarrhoea [51%] were reported as the symptoms with the largest impact on QoL.^14^ We only identified one study that reported an association between the presence of symptoms [rectal bleeding, stool frequency, urgency, general wellbeing, extracolonic features] and the risk of relapse in patients in clinical remission.^15^ There was little published evidence about the thresholds for symptoms or the PROMs used to measure these symptoms that would indicate clinical remission and could therefore be used to define comprehensive disease control [Table 2].

**Table 2.** PROMs included in statements.

PROM	Description	Validation or evidence of association
**Rectal bleeding**		
**PRO2 and pMCS: rectal bleeding domain**	1 item, 4 response options	The most recent FDA and EMA guidance for developing drugs for IBD do not recognise any fully validated/reliable tools for scoring the signs and symptoms of UC, including the PRO2 and pMCS^42,43^
**Urgency**		
**Urgency NRS**	1 item, 11-point scale [0–10], 24-h recall^44^	2-week diary study of 41 patients with UC found that the urgency NRS demonstrated high test–retest reliability, construct validity [high to moderate correlation with Patient Global Rating of Severity score], and content validity^44^
**Stool frequency/diarrhoea**		
**PRO2 and pMCS: Stool frequency domain**	1 item, 4 response options	The most recent FDA and EMA guidance for developing drugs for IBD do not recognise any fully validated/reliable tools for scoring the signs and symptoms of UC, including the PRO2 and pMCS^42,43^
**Abdominal pain**		
**VAS**	VAS [10-cm scale]	A study of 150 patients with UC, of whom 84 were in remission, used a VAS to evaluate pain and found a positive correlation between VAS score and endoscopic activity, as assessed using the MES^45^
**Fatigue**		
**IBD-F [SCORE 1 component]**	Evaluates level and duration of fatigue—5 questions, 0–4 scale, 2-week recall^46,47^	• Demonstrated good face and content validity, acceptable to excellent test–retest stability, and a high degree of internal consistency^48^ • In a study of 157 patients, the IBD-F demonstrated a strong correlation with HRQoL, as assessed using the SIBDQ [*p* <0.001]^47^ ◦ In this study, a score of 7.5 discriminated between significant and non-significant fatigue, as defined using the generic FSS PROM, with a specificity of 79.6% and a sensitivity of 73.7%^47^ • Patients have reported a preference for the IBD-F scale compared with other generic fatigue scales^48^
**FACIT-F**	13 items, 5-point response, not at all to very much, 7-day recall	Validated in 209 patients with IBD, with FACIT-F scores shown to correlate with inflammatory biomarkers of disease activity [CRP, ESR, and haematocrit]^49^
**Disease-related QoL**		
**SIBDQ**	10 questions, 7-point scale [all of the time to none of the time], 2-week recall^50^	• Published SLRs have shown that the SIBDQ has good consistency, reliability, and validity, and has been shown to match clinical/biological response in clinical trials as a secondary outcome^40^ • Correlations were seen with the IBD disability index and SIBDQ scores^51^
**IBD-Disk**	10 aspects, 0–10 scale [absolutely disagree to absolutely agree], 1-week recall	• In a study of 127 patients with UC, the IBD-Disk study demonstrated good correlation with the IBD disability index, and excellent reproducibility [intraclass correlation coefficient = 0.90] and internal consistency [Cronbach’s α = 0.89]^52^ ◦ The IBD-Disk was also shown to be associated with clinical disease activity^52^ • In a study of 40 patients with IBD, 30% of whom had UC, the internal consistency was high with significant differences seen between patients with and without disease activity^51^ ◦ This study proposed a threshold of 0–24 to indicate no disability, 25–49 for mild disability, 50–74 for moderate disability, and 75–100 for severe disability^51^

Note, PROMs were identified via a TLR and were not identified systematically.

CRP, C-reactive protein; EMA, European Medicines Agency; ESR, erythrocyte sedimentation rate; FACIT-F, Functional Assessment of Chronic Illness TherapyFatigue; FDA, Food and Drug Administration; FSS, Fatigue Severity Scale; HRQoL, health-related quality Of life; IBD, inflammatory bowel disease; IBD-Disk; inflammatory bowel disease-Disk; IBD-F, inflammatory bowel disease-fatigue; MES, Mayo endoscopic score; NRS, numerical rating scale; pMCS, partial Mayo Clinic Score; PRO2, 2-item patient-reported outcome; PROM, patient-reported outcome measure; QoL, quality of life; SIBDQ, Short Inflammatory Bowel Disease Questionnaire; SLR, systematic literature review; TLR, targeted literature review; UC, ulcerative colitis; VAS, visual analogue scale.

#### 3.1.3. Delphi consensus

During the first voting round, the panel ranked rectal bleeding as the most important symptom, followed by urgency and stool frequency/diarrhoea. When considering extraintestinal manifestations, joint pain was voted as the most important, followed by uveitis, skin symptoms, venous vascular comorbidities, and finally pulmonary involvement.

Over the three voting rounds, the panel agreed that comprehensive disease control should include the assessment of the following symptoms of UC, as reported by patients: rectal bleeding, stool frequency, bowel urgency, abdominal pain, extraintestinal manifestations, fatigue, and sleep disturbance. The panel also agreed that disease-related QoL should be included.

The panel agreed that the PRO2/pMCS can be used to measure rectal bleeding and stool frequency, and that the threshold for comprehensive disease control should be a rectal bleeding subscore of 0 and a stool frequency subscore of 1 or less [indicating 1–2 stools or fewer more than is normal for the person]. When looking at how to measure other symptoms, the panel agreed that a numerical scale can be used to assess the severity and frequency of urgency [eg, urgency numerical rating scale], abdominal pain [eg, a visual analogue scale], fatigue [eg, IBD-F/IBD-F SCORE 1 component or the FACIT-F], and sleep disturbance [eg, the PROMIS sleep disturbance item bank]. A simple system [ie, none, mild, moderate, or severe] can also be used to assess abdominal pain and fatigue. When considering thresholds, the panel agreed that urgency and abdominal pain should be absent when defining comprehensive disease control, with the exception of mild urgency, if patients do not find this impactful. For fatigue and sleep disturbance, the panel agreed that comprehensive disease control should be characterised by meaningful reductions in these symptoms for patients who had impactful fatigue or sleep disturbance while their UC was active [although these symptoms should be excluded if not related to UC].

To evaluate disease-related QoL, the panel agreed that the SIBDQ or the inflammatory bowel disease [IBD]-Disk should be used, which are simplified versions of the IBD questionnaire and IBD-Disability Index that were designed to be easier to complete than the originals. A threshold of no disability or IBD-Disk score of 24 or less was agreed. We did not identify any validated thresholds for the SIBDQ that would indicate remission in the literature, and so we did not suggest a specific threshold for the SIBDQ.

When looking at the strength of the recommendation, the PRO with the least agreement as shown by the mean score on the six-point Likert scale, that still met consensus, was sleep disturbance, with the statement about its importance only achieving a mean score of 3.8 out of 6 [with higher scores indicating stronger agreement with the statement]. Only in voting Round 3, after extensive discussion during the live meeting, was consensus reached to include sleep disturbance in a measure of comprehensive disease control. During the live meeting, the panel voiced concerns that sleep disturbance can result from other causes, and a complete absence may not be possible to achieve. Therefore, a threshold that excluded sleep disturbance from other causes, and that focused on meaningful reduction rather than complete absence, was agreed.

### 3.2. Clinical measures of inflammation

The panel agreed that a measure of comprehensive disease control should include endoscopic remission, inflammatory biomarkers, and histology. The panel also agreed that ultrasound [and other imaging techniques] can assess mucosal healing and is important to consider when evaluating comprehensive disease control, but did not recommend that ultrasound should be used at this time. The literature reviews informed which symptoms were included in the Delphi statements [Table 3]. Table 4 includes information on the tools and thresholds recommended to assess objective measures of disease activity.

**Table 3.** Rationale for including aspects in statements [ranked by order of importance, as voted on by panel].

Aspect	Results from literature reviews
**Endoscopic remission**	• Aligns with STRIDE II on the inclusion of endoscopic remission healing in a measure of remission • The SLR showed reduced risk of relapse in patients with endoscopic remission [UCEIS or MES ≤ 1]
**Inflammatory biomarkers**	• Aligns with STRIDE II on the inclusion of inflammatory biomarkers in a measure of remission • The SLR found several studies showing a strong association between FC and CRP and clinical disease activity and the risk of relapse
**Histology**	• Not recommended by STRIDE II—there is no additional evidence available since STRIDE II that suggests including histological healing in a measure of remission • The SLR indicated that there is still uncertainty in the association between histological healing and clinical outcomes
**Ultrasound**	• Endoscopy can be invasive and resource intensive; ultrasound provides a less invasive method of monitoring endoscopic disease activity and can therefore be performed more frequently than endoscopy

CRP, C-reactive protein; FC, faecal calprotectin; MES, Mayo endoscopic score; SLR, systematic literature review; STRIDE II, Selecting Therapeutic Targets in Inflammatory Bowel Disease II; UCEIS, Ulcerative Colitis Endoscopic Index of Severity.

**Table 4.** Measures of inflammation included in statements.

Tool	Validation or evidence of association
**Endoscopic remission**	
**MES**	• Limited validation and may be subject to inter-observer disagreement^53^ • Less complex than the UCEIS and therefore more widely used^17^ • In our SLR, there was no difference in clinical outcomes between patients with MES or UCEIS score of 0 vs 1, indicating that targeting a more stringent threshold of 0 compared with ≤1 has no clinical benefit
**UCEIS**	• Limited validation and may be subject to inter-observer disagreement^53^ • Undergone a rigorous development process, considers objective items, and has a strong prognostic value^17^ **•** In our SLR, there was no difference in clinical outcomes between patients with UCEIS score of 0 vs 1, indicating that targeting a more stringent threshold of 0 compared with ≤1 has no clinical benefit
**Ultrasound**	
**UC-IUS index**	• A study of 60 patients found that UC-IUS was strongly correlated with MES^54^ ◦ A cut-off of 3.2 mm was optimal to discriminate between MES ≤1 and MES 2–3 [sensitivity 89.1%; specificity 92.3%; AUC 0.946]^54^

Note, measures of inflammation were identified via a targeted literature review and were not identified systematically.

AUC, area under the curve; MES, Mayo endoscopic score; SLR, systematic literature review; UCEIS, Ulcerative Colitis Endoscopic Index of Severity; UC-IUS, Ulcerative Colitis-Intestinal Ultrasound.

#### 3.2.1. Endoscopic remission

**Table:** 

Final statements
**Endoscopic remission is important to consider when assessing comprehensive disease control** Consensus: Round 1; 9/9 agreed [100%], strength^a^ 5.6 [5–6]
**Endoscopic remission should be included in a measure of comprehensive disease control** Consensus: Round 1, 9/9 agreed [100%]; strength^a^ 5.7 [6]
**The Mayo endoscopic score [MES] and the UC Endoscopic Index of Severity [UCEIS] should be used to assess endoscopic remission, using the threshold ≤1** Consensus: Round 1, 9/9 agreed [100%]; strength^a^ 5.2 [5–6]
**Is there enough evidence to suggest the best timing of endoscopy and evaluate whether response-guided assessments are more appropriate than performing endoscopies at fixed intervals?** Consensus: Round 1, 6/8^b^ voted No [75%]; strength^a^ N/A^c^

a Strength of recommendation: mean and IQR of six-point Likert scale response.

b One panel member did not answer this question because it was beyond their professional knowledge.

c Responses were not provided on a six-point Likert scale in voting Round 3.

##### 3.2.1.1. Pre-Delphi research

Studies identified by our SLR and TLRs indicated that endoscopic remission is associated with improved clinical outcomes, with patients in endoscopic remission shown to have fewer relapses compared with those not in remission.^15,16^ When looking at the most appropriate tool to score endoscopic remission, the MES and the UCEIS were the most widely used scores [although note that this was not a specific objective of the SLR]. The UCEIS has undergone a more rigorous development than the MES but is more complicated to use and is used less frequently in clinical practice.^17^

When considering the threshold for comprehensive disease control, it is unclear whether depth of endoscopic remission impacts on clinical outcomes. Evidence identified from STRIDE II showed that patients with an MES of 0 had improved outcomes compared with those with an MES of 1.^18–20^ In contrast, published evidence identified by our SLR indicated that, for patients in clinical remission, there were no differences in relapse outcomes between those with MES or UCEIS scores of 0 versus 1 or less.^21–23^

##### 3.2.1.2. Delphi consensus

The panel agreed that endoscopic remission should be included in a measure of comprehensive disease control and that the MES and UCEIS should be used, agreeing a threshold score of 1 or less for endoscopic remission.

#### 3.2.2. Inflammatory biomarkers

**Table:** 

Final statements
**Inflammatory biomarkers provide a non-invasive measure that can be used to monitor patients and should be performed regularly to allow early detection of disease activity** Consensus: Round 1, 9/9 agreed [100%]; strength^a^ 5.7 [5–6]
**Inflammatory biomarkers should be included in a measure of comprehensive disease control** Consensus: Round 1, 9/9 agreed [100%]; strength^a^ 5.7 [5–6]
**The threshold for remission for faecal calprotectin [FC] should be ≤100–250 μg/g, and the threshold for CRP should be below the upper limit of normal** Consensus: Round 1, 8/9 agreed [88.9%]; strength^a^ 4.9 [5]
**FC and CRP levels should be given equal weighting when assessing remission** Consensus: Round 1, 8/9 disagreed [88.9%]; strength^a^ 2.3 [2–3]
**FC levels should be prioritised over CRP levels when assessing remission** Consensus: Round 1, 9/9 agreed [100%]; strength^a^ 5.3 [5–6]
**CRP levels should be prioritised over FC levels when assessing remission** Consensus: Round 1, 7/9 disagreed [77.8%]; strength^a^ 2.4 [1–3]

a Strength of recommendation: mean and IQR of six-point Likert scale response.

b Responses were not provided on a six-point Likert scale in voting Round 3.

##### 3.2.2.1. Pre-Delphi research

In the pre-voting survey, nine out of 10 gastroenterologists reported that they regularly evaluated FC and/or CRP levels as part of routine care, at intervals of 2–12 months. The remaining gastroenterologist reported that evaluation of FC and CRP was not reimbursed for UC in their country. Published findings show an association between FC and/or CRP levels and clinical outcomes.^24,25^ The thresholds for these biomarkers reported in the literature and during the pre-voting survey are displayed in Supplementary Table 8.

##### 3.2.2.2. Delphi consensus

The panel agreed that inflammatory biomarkers should be evaluated as part of comprehensive disease control, with a threshold for FC of 100–250 μg/g or less and a threshold for CRP below the upper limit of normal. The panel agreed that FC levels should be prioritised over CRP levels when assessing comprehensive disease control.

#### 3.2.3. Histology

**Table:** 

Final statements
**Histology provides information on inflammatory disease activity** Consensus: Round 1, 9/9 agreed [100%]; strength^a^ 5.2 [5]
**Histological inflammatory activity should be absent for remission to be considered comprehensive** Consensus: Round 3, 9/9 agreed [100%]; strength^a^ N/A^b^

a Strength of recommendation: mean and IQR of six-point Likert scale response.

b Responses were not provided on a six-point Likert scale in voting Round 3.

##### 3.2.3.1. Pre-Delphi research

Some published studies report that histological-endoscopic remission is a greater predictor of long-term remission than endoscopic remission alone.^9,26,27^ However, other published studies showed no clear association between histological remission and clinical outcomes.^21,28^ Consequently, the relationship between histology and clinical outcomes is unclear.

##### 3.2.3.2. Delphi consensus

The panel recommended that histological inflammatory activity should be absent in our definition of comprehensive disease control. No specific scoring tools or threshold were identified, highlighting an area for future research.

#### 3.2.4. Ultrasound and other imaging techniques

**Table:** 

Final statements
**Ultrasound and other imaging techniques can assess mucosal healing and are important to consider when assessing comprehensive disease control** Consensus: Round 1, 6/8 agreed [75.0%]^a^; strength^b^ 4.3 [4–5]
**Ultrasound results should be assessed using the Ulcerative Colitis-Intestinal Ultrasound [UC-IUS] index** Consensus: Round 1, 7/9 agreed [77.8%]; strength^b^ 4.1 [4–5]
**The threshold for remission using ultrasound should be bowel wall thickness ≤3.2 mm** Consensus: Round 1, 7/9 agreed [77.8%]; strength^b^ 4.1 [4–5]

a Strength of recommendation: mean and IQR of six-point Likert scale response.

b One panel member did not answer this question because it was beyond their professional knowledge.

Owing to the invasiveness and cost associated with endoscopy, there is interest as to whether there are less invasive imaging techniques that can be used to evaluate endoscopic activity. Indeed, the use of gastrointestinal ultrasound to monitor endoscopic activity has been increasing in recent years.^29^ However, the panel highlighted that ultrasound is still not widely available in many countries and treatment settings. Our combined definition of comprehensive disease control is intended for immediate use in clinical practice; therefore, we did not fully evaluate the literature around ultrasound but included it in the statements for completeness.

##### 3.2.4.1. Delphi consensus results

The panel agreed that ultrasound and other imaging techniques can be used to assess mucosal healing and are important to consider when assessing comprehensive disease control. However, they stopped short of including ultrasound in a measure of comprehensive disease control at this time because of the lack of availability in many countries and centres.

### 3.3. Treatments during remission

**Table:** 

Final statements
**Discontinuation of corticosteroids is important to consider when assessing comprehensive disease control** Consensus: Round 1, 9/9 agreed [100%]; strength^a^ 5.9 [6]
**Discontinuation of azathioprine is important to consider when assessing comprehensive disease control** Consensus: Round 1, 7/9 disagreed [77.8%]; strength^a^ 2.4 [2–3]
**Discontinuation of 5-aminosalicylic acid [5-ASA] is important to consider when assessing comprehensive disease control** Consensus: Round 1, 7/9 disagreed [77.8%]; strength^a^ 2.0 [1–3]
**Discontinuation of corticosteroids should be included in a measure of comprehensive disease control** Consensus: Round 1, 9/9 agreed [100%]; strength^a^ 5.8 [6]
**Discontinuation of azathioprine should be included in a measure of comprehensive disease control** Consensus: Round 1, 7/9 disagreed [77.8%]; strength^a^ 2.2 [1–2]
**Discontinuation of 5-ASA should be included in a measure of comprehensive disease control** Consensus: Round 1, 7/9 disagreed [77.8%]; strength^a^ 2.0 [1–2]
**Discontinuation of biologics or small molecules is important to consider when assessing comprehensive disease control** Consensus: Round 2, 9/9 disagreed [100%]; strength^a^ 2.0 [2]
**Discontinuation of biologics or small molecules should be included in a measure of comprehensive disease control** Consensus: Round 2, 8/9 disagreed [88.9%]; strength^a^ 2.1 [2]

a Strength of recommendation, mean and IQR of six-point Likert scale response.

#### 3.3.1.1. Pre-Delphi research

Corticosteroid-free remission is often used as a treatment target for UC.^30^ Patients can also receive other therapies during remission, such as 5-ASAs, azathioprine, biologics, and small molecules, which may lead to side effects that affect patient wellbeing; for example, 10–28% of patients report adverse events with azathioprine, and continual use of thiopurines is linked to an increased risk of malignancies such as lymphoma and non-melanoma skin cancer.^31^ The burden of continuous therapy during remission has not been well evaluated, although treatments with a high ‘pill burden’ may affect patients’ QoL.^32^

#### 3.3.1.2. Delphi consensus results

There was strong consensus that discontinuation of corticosteroids is important and should be included in a measure of comprehensive disease control. There was also consensus that discontinuing other therapies, including 5-ASA, azathioprine, and biologics, was considered not important and was therefore not included in a measure of comprehensive disease control.

## 4. Discussion

We recommend that the following aspects of UC should be considered in combination when defining comprehensive disease control: rectal bleeding, stool frequency, bowel urgency, abdominal pain, extraintestinal manifestations, disease-related QoL, endoscopy, histological inflammatory activity, inflammatory biomarkers, use of corticosteroids, fatigue, and sleep disturbance. We have recommended tools or scoring systems for many of these aspects and have suggested threshold scores for assessing whether patients with UC have comprehensive disease control in some cases. By acknowledging the need to incorporate symptoms that are important to patients, we are closer to trying to comprehensively regain the mental, physical, and emotional health of patients with UC.

This work agrees on a standard set of outcomes that should be measured and reported, both in clinical trials and in real-world clinical practice,^33^ covering aspects important to the daily life of patients and to the future course of UC. As such, it can also be thought of as the first step towards developing a core outcome set to evaluate comprehensive disease control in trials and clinical practice in UC. Our next step is to fully combine the measurement and scoring of these aspects into a multicomponent tool, noting that the sleep disturbance and fatigue questionnaires mentioned in the statements would require simplification to allow for pragmatic use in the clinic. In addition, this tool will require validation with patients in prospective treatment settings, including validation of thresholds to indicate comprehensive disease control. The feasibility of using this multicomponent measure also needs evaluating in clinical practice because, although we have recommended measures partly based on the ease and simplicity of completion, these measures need assessing in composite.

These recommendations have been made following a robust and well-recognised methodology that included patients from the start of the process. By surveying patients, we were able to understand which aspects of UC were most important to them while in remission, supplemented by our review of published literature. This was combined with the experience of our panellists—gastroenterologists with expertise in practice and clinical trials—plus our patient advocate on the panel who spoke to the patient experience. Panellists were surveyed on their thoughts on aspects of UC that are important in remission, were provided with summaries of relevant recent literature, and voted on statements over three voting rounds. An additional reviewer with specific expertise in PROMs reviewed the statements prior to voting, focusing on methodological aspects and feasibility of PROM use in practice. Comparing the differences between the patient and physician surveys highlighted an initial greater tolerance of symptoms among the physicians. Indeed, the consensus statements that progressed to later voting rounds concerned levels of symptoms during remission. The majority of statements that reached strong consensus in voting Round 1 included aspects previously agreed in STRIDE II, such as rectal bleeding, blood in stools, QoL, endoscopy, and inflammatory biomarkers.

To put this work in the context of existing work, we note that our Delphi built on the recommendations from the STRIDE II initiative.^9^ We aligned with STRIDE II on the evaluation of rectal bleeding and stool frequency, as measured using the PRO2, and the inclusion of QoL, but we also included additional patient-reported symptoms that we found to be of high importance to patients in our survey, namely bowel urgency, abdominal pain, extraintestinal manifestations, fatigue, and sleep disturbance. We recommended the same threshold for rectal bleeding as STRIDE II [rectal bleeding subscore = 0], but a less stringent threshold for stool frequency [stool frequency subscore = 1, indicating 1–2 stools more than is normal for them]. Similar to STRIDE II, the panel agreed to include endoscopic remission and inflammatory biomarkers, but also included inflammatory histological activity, which was not recommended as a specific treatment goal in STRIDE II. Our suggested threshold for endoscopic remission was less stringent than that suggested in STRIDE II because evidence identified by our SLR seemed to indicate that the depth of endoscopic remission [ie, MES/UCEIS score of 0 vs ≤1] did not affect clinical outcomes.^21–23,34^ However, there are conflicting findings, with some studies demonstrating that patients with an MES or UCEIS score of 0 had improved outcomes compared with those with a score of 1;^18–20,35^ therefore, this remains an area for further investigation.

In addition to STRIDE II, other initiatives have evaluated outcomes in UC/IBD. The CORE-IBD^11^ and Health Outcomes Observatory [H2O]^36^ projects both used a Delphi process to create core outcome sets for evaluating remission and included a larger number of patients and experts on the panel than we included [CORE-IBD: 235 patients and 53 experts; H2O: 45 patients and 91 experts]. The CORE-IBD initiative core outcome set is designed for clinical trials, and the H2O project is designed for clinical practice; in contrast, we have defined a measure that can be implemented in both clinical trials and clinical practice. The CORE-IBD initiative recommended assessing urgency and histology, but it did not include additional patient-reported symptoms,^11^ making it less ‘comprehensive’ than this current work. The H2O initiative did include more PROs, but no further detail was available on what these were.^36^ The CORE-IBD initiative was the only initiative that provided a definition of remission and suggested thresholds. However, they did not include urgency in their definition of symptomatic remission; it was instead included in a multicomponent PRO index, alongside stool frequency and rectal bleeding, and so it was less comprehensive than our measure of disease control. Finally, a literature review by Wetwittayakhlang *et al.* evaluated treatment targets for use in clinical practice and proposed treatment targets similar to those recommended by STRIDE II, similarly mentioning histology as an important potential therapeutic goal but with uncertainty as to whether it should be incorporated into routine clinical practice.^37^

Limitations of our approach include those related to remission of UC itself—we do not know if patients will reach the suggested thresholds across the aspects included, to fulfil the definition of comprehensive disease control. This also reflects the individualised nature of the assessments—for example, the level of urgency and abdominal pain that a patient can experience without it affecting them will differ between patients—hence the level of symptoms consistent with comprehensive disease control will differ between patients.

Another limitation is that our panellists and most of our patients were from Europe and do not, therefore, reflect differences in clinical practice between Europe and the rest of the world, including in the procedures and evaluations used for UC. Where clear differences exist within Europe for example, access to ultrasound [as highlighted by the panel] this was reflected in the final statements, but other differences, such as reimbursement levels for testing inflammatory biomarkers or familiarity with certain evaluations, were not covered. Another limitation related to geography is that there may be differences in how PROMs are perceived by patients from different cultural and social backgrounds, and this would need addressing during validation. A key limitation is that relatively few patients completed the survey [*n* = 18], and we cannot say with confidence that these patients were representative of the global population of patients with UC. In addition, only one patient expert took part in the Delphi panel, although they did vote in each round and fully contributed at the live meeting. In the consensus voting rounds, the threshold of 67% or higher of participants agreeing provided a binary response; however, we also used a six-point Likert scale to understand the strength of agreement. A final limitation is that our literature review of PROMs was not systematic, meaning that we may have missed some validation or threshold studies, and the PROMs included in the statements were not a complete list of PROMs that may be suitable. For example, the IBD-Control questionnaire can also be used to measure disease-related QoL and has been designed and validated for use in routine clinical practice.^38^

## 5. Conclusion

We recommend aspects of UC that should be included in a combined measure of comprehensive disease control, which is patient-centric and should have applicability in the real-world individualised treatment of patients, as well as in evaluating treatment efficacy in clinical trials. Future work will evaluate the feasibility and validity of developing this as a multicomponent tool for ease of use. Defining comprehensive disease control could be an important step towards individualied treatment in UC and a treatment target in UC.

## Supplementary Material

jjad130_suppl_Supplementary_Data

jjad130_suppl_Supplementary_Materials

## Data Availability

All data are incorporated into the article and its online Supplementary materials.
