# Supplementary material for: Defining Comprehensive Disease Control for Use as a Treatment Target for Ulcerative Colitis in Clinical Practice: International Delphi Consensus Recommendations
Source: J Crohns Colitis. 2023 Aug 16;18(1):91–105. doi: 10.1093/ecco-jcc/jjad130 (PMC10821705; doi:10.1093/ecco-jcc/jjad130)
Supplement: jjad130_suppl_Supplementary_Data [file jjad130_suppl_supplementary_data.docx]

­­­­Defining Comprehensive Disease Control for use as a Treatment Target for Ulcerative Colitis in Clinical Practice: International Delphi Consensus Recommendations

# Supplementary materials

## Patient survey – additional methodology

For the patient survey, we recruited patients with UC who had experience of remission, mainly through our professional and personal network, and contacted relevant patient organisations. Anyone interested was emailed details of the study, its objectives, and information on how their data would be used. Participants were reimbursed for their time with a voucher equivalent to £25.

### Patient questionnaire

## Systematic and targeted reviews – additional methodology

The systematic literature review (SLR) identified evidence for adult patients with UC in clinical remission reporting:

1. endoscopy or histology assessment or inflammatory biomarker levels, if they evaluated correlation or association with a clinical event, a patient-reported outcome measure (PROM), or an established measurement of disease activity; or
2. studies using any type of PROM (symptoms, complaints, treatment satisfaction/preference, burden of treatment, adverse events, QoL, or work productivity), if they evaluated correlation or association with another outcome or validity assessment of the PROM.

Electronic databases (Embase, MEDLINE, and the Cochrane Library) were searched from 1 January 2019, to capture evidence published since the searches for STRIDE II were conducted in July 2019, to 22 March 2022. We also searched key congresses shown in Supplementary Table 1. The SLR search strings included terms for UC, remission, PROMs, and objective measures of disease activity (see Supplementary Table 2, Supplementary Table 3, and Supplementary Table 4 for the full search strings). Studies that met the predefined inclusion/exclusion criteria detailed in Supplementary Table 5 were included. The protocol was registered with PROSPERO.^1^

## Delphi process – additional methodology

In the Delphi process, a small number of questions had ‘yes’ or ‘no’ responses, to determine whether an aspect should be considered in subsequent voting rounds. For example, whether the panel thought there was enough evidence to consider the most appropriate timing of endoscopy. In case of disagreement, free text was used to provide comments. In addition, the panel ranked symptoms and clinical measures by importance. Where there were two statements providing alternative suggestions (for example, on how to measure a symptom), if one statement met consensus and the other did not, the statement that did not meet consensus was discarded. Some statements were also superseded following the virtual meeting and final voting round.

### Pre-voting questionnaires

## Supplementary tables and figures

Supplementary Table 1. Congresses searched.

| **Congress** | **Years to be searched*** |
| --- | --- |
| United European Gastroenterology Week: <https://ueg.eu/week> | 2019*, 2020, 2021 |
| Digestive Disease Week: <https://ddw.org/> | 2019*, 2020, 2021 |
| European Crohn’s and Colitis Organisation: <https://www.ecco-ibd.eu/ecco22.html> | 2019*, 2020, 2021 |
| Advances in Inflammatory Bowel Diseases: <https://www.advancesinibd.com/> | 2019*, 2020, 2021 |
| ACG Annual Scientific Meeting: <https://acgmeetings.gi.org/annual-meeting/> | 2019*, 2020, 2021 |

*In line with the date of the STRIDE II update, we covered congresses from July 2019.

Supplementary Table 2. Embase searches, ran 22 March 2022.

| **#** | **Searches** | **Results** |
| --- | --- | --- |
| 1 | exp ulcerative colitis/ | 82660 |
| 2 | ulcerative colitis.mp. | 91787 |
| 3 | 1 or 2 | 91787 |
| 4 | remission/ | 237126 |
| 5 | remission*.ti,ab. | 228670 |
| 6 | 4 or 5 | 338534 |
| 7 | (((index or scale* or score*) and clinic*) not (microscopic colitis or mastocytosis or cancer or irritable)).mp. | 2043097 |
| 8 | exp patient-reported outcome/ or exp "Quality of Life"/ or (Patient reported outcome* or self-reported outcome* or QoL or hrql or hrqol or quality of life or fatigue or disability or productivity or depression or anxiety).mp. | 2169168 |
| 9 | ((endoscop* or colonoscop* or sigmoidoscop* or proctosigmoidoscop* or endoscopic) and (index or score* or grade* or Baron or Rachmilewitz or Mayo or Matts or UCEIS or Truelove or Dick or Marks or Feagan or Powell or Lemann or Sutherland or healing)).mp. | 99735 |
| 10 | (mucosal healing or histological healing or histological scoring).mp. | 6896 |
| 11 | (imaging or ultrasound or magnetic resonance or computed tomography).mp. | 3199223 |
| 12 | ((calprotectin or lactoferrin or C-reactive protein or erythrocyte sedimentation rate* or hemoglobin or bone mineral density or ferritin or prostaglandin) and (response or remission or flare)).mp. | 169040 |
| 13 | or/7-12 | 6717774 |
| 14 | Clinical Trial/ or Randomized Controlled Trial/ or controlled clinical trial/ or multicenter study/ or Phase 1 clinical trial/ or Phase 2 clinical trial/ or Phase 3 clinical trial/ or Phase 4 clinical trial/ or exp RANDOMIZATION/ or Single Blind Procedure/ or Double Blind Procedure/ or Crossover Procedure/ or PLACEBO/ or randomi?ed controlled trial$.tw. or rct.tw. or (random$ adj2 allocat$).tw. or single blind$.tw. or double blind$.tw. or ((treble or triple) adj blind$).tw. or placebo$.tw. or Prospective Study/ | 2706204 |
| 15 | Clinical study/ | 157579 |
| 16 | Case control study/ | 185674 |
| 17 | Family study/ | 25397 |
| 18 | Longitudinal study/ | 169650 |
| 19 | Retrospective study/ | 1218698 |
| 20 | Prospective study/ | 753973 |
| 21 | Randomized controlled trials/ | 222816 |
| 22 | 20 not 21 | 745246 |
| 23 | Cohort analysis/ | 820617 |
| 24 | (Cohort adj (study or studies)).mp. | 392492 |
| 25 | (Case control adj (study or studies)).tw. | 152389 |
| 26 | (follow up adj (study or studies)).tw. | 68742 |
| 27 | (observational adj (study or studies)).tw. | 212413 |
| 28 | (epidemiologic$ adj (study or studies)).tw. | 115038 |
| 29 | (cross sectional adj (study or studies)).tw. | 281694 |
| 30 | (registry or register$ or survey).ti,ab. | 1268929 |
| 31 | (real world or RWE).ti,ab. | 101366 |
| 32 | Real-life.ti,ab. | 40885 |
| 33 | or/15-19,22-32 | 4425512 |
| 34 | 3 and 6 and 13 and (14 or 33) | 5682 |
| 35 | (animal$ not human$).sh,hw. | 4613569 |
| 36 | 34 not 35 | 5654 |
| 37 | case study/ or case report.tw. | 561753 |
| 38 | 36 not 37 | 5561 |
| 39 | limit 38 to english language | 5484 |
| 40 | limit 39 to yr="2019 -Current" | 2010 |
| 41 | remove duplicates from 40 | 1985 |
| 42 | conference abstract.pt. | 4353293 |
| 43 | 41 not 42 | 833 |

Supplementary Table 3. Medline searches, run on 10 March 2022.

| **#** | **Searches** | **Results** |
| --- | --- | --- |
| 1 | exp ulcerative colitis/ | 37962 |
| 2 | ulcerative colitis.mp. | 44853 |
| 3 | 1 or 2 | 54078 |
| 4 | remission*.ti,ab. | 136541 |
| 5 | (((index or scale* or score*) and clinic*) not (microscopic colitis or mastocytosis or cancer or irritable)).mp. | 839222 |
| 6 | exp patient-reported outcome/ or exp "Quality of Life"/ or (Patient reported outcome* or self-reported outcome* or QoL or hrql or hrqol or quality of life or fatigue or disability or productivity or depression or anxiety).mp. | 1352106 |
| 7 | ((endoscop* or colonoscop* or sigmoidoscop* or proctosigmoidoscop* or endoscopic) and (index or score* or grade* or Baron or Rachmilewitz or Mayo or Matts or UCEIS or Truelove or Dick or Marks or Feagan or Powell or Lemann or Sutherland or healing)).mp. | 48964 |
| 8 | (mucosal healing or histological healing or histological scoring).mp. | 3203 |
| 9 | (imaging or ultrasound or magnetic resonance or computed tomography).mp. | 2696295 |
| 10 | ((calprotectin or lactoferrin or C-reactive protein or erythrocyte sedimentation rate* or hemoglobin or bone mineral density or ferritin or prostaglandin) and (response or remission or flare)).mp. | 68278 |
| 11 | or/5-10 | 4608286 |
| 12 | Randomized Controlled Trials as Topic/ or randomized controlled trial/ or Random Allocation/ or Double Blind Method/ or Single Blind Method/ or clinical trial/ or clinical trial, phase i.pt. or clinical trial, phase ii.pt. or clinical trial, phase iii.pt. or clinical trial, phase iv.pt. or controlled clinical trial.pt. or randomized controlled trial.pt. or multicenter study.pt. or clinical trial.pt. or exp Clinical Trials as topic/ or (clinical adj trial$).tw. or ((singl$ or doubl$ or treb$ or tripl$) adj (blind$3 or mask$3)).tw. or PLACEBOS/ or placebo$.tw. or randomly allocated.tw. or (allocated adj2 random$).tw. | 1809206 |
| 13 | Epidemiologic studies/ or exp case control studies/ or exp cohort studies/ or Case control.tw. or (cohort adj (study or studies)).tw. or Cohort analy$.tw. or (Follow up adj (study or studies)).tw. or (observational adj (study or studies)).tw. or Longitudinal.tw. or Retrospective.tw. or Cross sectional.tw. or Cross-sectional studies/ or (registry or register$ or survey).ti,ab. or (real world or RWE).ti,ab. or Real-life.ti,ab. | 4148211 |
| 14 | 3 and 4 and 11 and (12 or 13) | 1926 |
| 15 | (animal$ not human$).sh,hw. | 4932352 |
| 16 | 14 not 15 | 1921 |
| 17 | case study/ or case report.tw. | 2314092 |
| 18 | 16 not 17 | 1907 |
| 19 | limit 18 to english language | 1848 |
| 20 | limit 19 to yr="2019 -Current" | 614 |
| 21 | remove duplicates from 20 | 611 |
| 22 | congress.pt. | 66981 |
| 23 | 21 not 22 | 611 |

Supplementary Table 4. Cochrane searches, run on 10 March 2022.

| **#** | **Searches** | **Results** |
| --- | --- | --- |
| 1 | exp ulcerative colitis/ | 1747 |
| 2 | ulcerative colitis.mp. | 5926 |
| 3 | 1 or 2 | 6102 |
| 4 | remission/ | 5 |
| 5 | remission*.ti,ab. | 27874 |
| 6 | 4 or 5 | 27879 |
| 7 | (((index or scale* or score*) and clinic*) not (microscopic colitis or mastocytosis or cancer or irritable)).mp. | 333672 |
| 8 | exp patient-reported outcome/ or exp "Quality of Life"/ or (Patient reported outcome* or self-reported outcome* or QoL or hrql or hrqol or quality of life or fatigue or disability or productivity or depression or anxiety).mp. | 294453 |
| 9 | ((endoscop* or colonoscop* or sigmoidoscop* or proctosigmoidoscop* or endoscopic) and (index or score* or grade* or Baron or Rachmilewitz or Mayo or Matts or UCEIS or Truelove or Dick or Marks or Feagan or Powell or Lemann or Sutherland or healing)).mp. | 16608 |
| 10 | (mucosal healing or histological healing or histological scoring).mp. | 964 |
| 11 | (imaging or ultrasound or magnetic resonance or computed tomography).mp. | 113639 |
| 12 | ((calprotectin or lactoferrin or C-reactive protein or erythrocyte sedimentation rate* or hemoglobin or bone mineral density or ferritin or prostaglandin) and (response or remission or flare)).mp. | 17361 |
| 13 | or/7-12 | 624511 |
| 14 | Randomized Controlled Trials as Topic/ or randomized controlled trial/ or Random Allocation/ or Double Blind Method/ or Single Blind Method/ or clinical trial/ or clinical trial, phase i.pt. or clinical trial, phase ii.pt. or clinical trial, phase iii.pt. or clinical trial, phase iv.pt. or controlled clinical trial.pt. or randomized controlled trial.pt. or multicenter study.pt. or clinical trial.pt. or exp Clinical Trials as topic/ or (clinical adj trial$).tw. or ((singl$ or doubl$ or treb$ or tripl$) adj (blind$3 or mask$3)).tw. or PLACEBOS/ or placebo$.tw. or randomly allocated.tw. or (allocated adj2 random$).tw. | 1003785 |
| 15 | Epidemiologic studies/ or exp case control studies/ or exp cohort studies/ or Case control.tw. or (cohort adj (study or studies)).tw. or Cohort analy$.tw. or (Follow up adj (study or studies)).tw. or (observational adj (study or studies)).tw. or Longitudinal.tw. or Retrospective.tw. or Cross sectional.tw. or Cross-sectional studies/ or (registry or register$ or survey).ti,ab. or (real world or RWE).ti,ab. or Real-life.ti,ab. | 339771 |
| 16 | 3 and 6 and 13 and (14 or 15) | 1323 |
| 17 | (animal$ not human$).sh,hw. | 2220 |
| 18 | 16 not 17 | 1323 |
| 19 | case study/ or case report.tw. | 2625 |
| 20 | 18 not 19 | 1321 |
| 21 | limit 20 to english language [Limit not valid in DARE,CLCMR,ACP Journal Club,CDSR; records were retained] | 987 |
| 22 | limit 21 to yr="2019 -Current" [Limit not valid in DARE; records were retained] | 292 |
| 23 | remove duplicates from 22 | 290 |
| 24 | conference abstract.pt. | 16837 |
| 25 | 23 not 24 | 290 |

Supplementary Table 5. Eligibility criteria for the SLR.

| **Eligibility criterion** | **Inclusion criteria** | **Exclusion criteria** |
| --- | --- | --- |
| Population | Adult UC in remission  Population ≥ 20 adults with UC in remission | Studies focusing on achieving remission (i.e. not reporting patients who are in remission).  Children with UC |
| Intervention/  Comparator | Any or none | N/A |
| Outcomes | For the following endpoints, include if reported with a correlation or an association with a clinical event, or a PROM or an established measurement of disease activity:   - endoscopy assessment/score - histology assessment/score - inflammatory biomarker levels     For the following endpoints, include if reported via a patient-reported outcome AND reported with a correlation or association with another outcome, i.e. clinical event, endoscopic, histologic, biomarkers OR the study investigates the validity of the PROM in the relevant population:   - residual symptoms - non-IBD related complaints - treatment satisfaction/preference - burden of treatment - adverse events - QoL - work productivity | If the correlation or association is not reported in the abstract, either as aims, methods, or results  Imaging assessments    If the correlation or association or investigation of validity is not reported in the abstract, either as aims, methods, or results |
| Study design | RCTs, single-arm trials, observational/real-world evidence studies (including long-term extension studies) | Animal/*in vitro* studies, case studies, and case reports  Congress abstracts |
| Date restrictions | 2019 to current | N/A |
| Language restrictions | English language | Non-English language |
| Publication type | All primary publications, SLRs meta-analyses, and indirect treatment comparisons^a^ | Non-SLRs, editorials, notes and letters |
| Country | Not restricted | N/A |

^a^SLRs, meta-analyses and indirect treatment comparisons reporting relevant study types will be listed without data extraction.

IBD, inflammatory bowel disease; QoL, quality of life; N/A, not applicable; PROM, patient-reported outcome measures; RCT, randomized controlled trial; SLR, systematic review; UC, ulcerative colitis.

Supplementary Figure 1. PRISMA flow diagram.


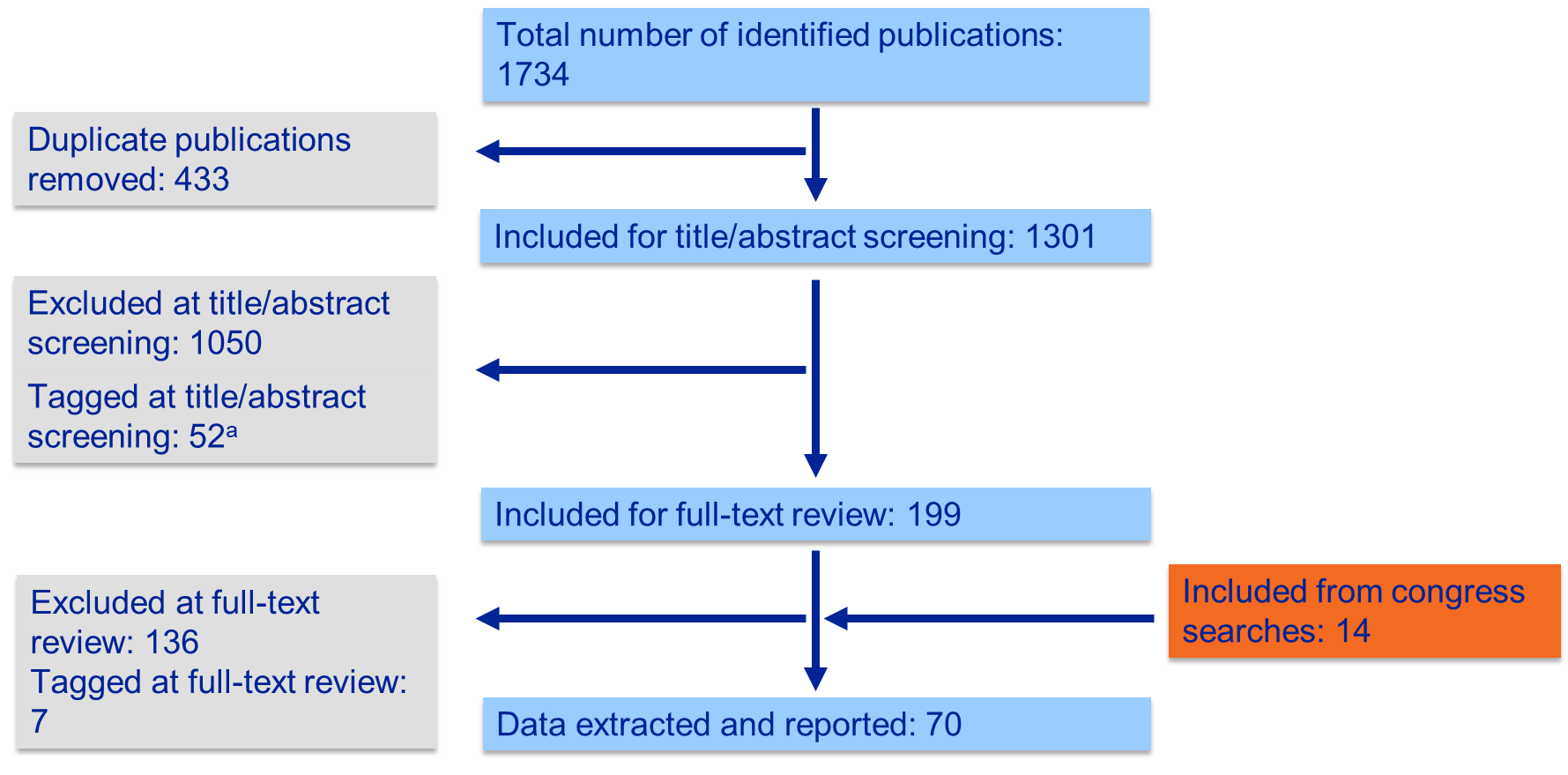


^a^RCTs and single-arm studies were tagged and not included.

PRISMA, Preferred Reporting Items for Systematic Reviews and Meta-Analyses; RCT, randomized controlled trial.

Supplementary Table 6. Characteristics of patients who completed the survey.

| **Characteristic, n (%)** | **Patients (N = 18)** |
| --- | --- |
| **Sex** |  |
| Male | 11 (61) |
| Female | 7 (39) |
| **Age** |  |
| 18–28 years | 4 (22) |
| 29–38 years | 8 (44) |
| 39–48 years | 2 (11) |
| 48–58 years | 2 (11) |
| ≥ 59 years | 2 (11) |
| **Time since diagnosis** |  |
| < 1 year | 0 (0) |
| 1–< 5 years | 1 (6) |
| 5–< 10 years | 6 (33) |
| 10–< 20 years | 7 (39) |
| ≥ 20 years | 4 (22) |
| **Time since last relapse** |  |
| < 1 year | 0 (0) |
| 1–< 2 months | 1 (6) |
| 3–< 6 months | 1 (6) |
| 6–< 12 months | 2 (11) |
| 12–< 24 months | 4 (22) |
| ≥ 24 months | 10 (56) |
| **Number of relapses in last 12 months** |  |
| 0 | 13 (72) |
| 1 | 1 (6) |
| 2 | 3 (17) |
| 3 | 0 (0) |
| ≥ 4 | 1 (6) |

Supplementary Table 7. All statements voted on and summary of changes between voting rounds.

| **Round 1** | | **Round 2** | | | **Round 3** | | |
| --- | --- | --- | --- | --- | --- | --- | --- |
| **Statement** | **Voting result** | **Statement** | **Voting result** | **Statement** | | **Voting result** | |
| ***Symptoms*** | | | | | | | |
| **Rectal bleeding** | | | | | | | |
| Rectal bleeding is important to consider when assessing comprehensive disease control. | 9/9 **agreed** (100%) | – | | | | | |
| Assessment of rectal bleeding should be included in a measure of comprehensive disease control. | 9/9 **agreed** (100%) | – | | | | | |
| The rectal bleeding component of PRO2/pMCS can be used when assessing the severity and frequency of rectal bleeding. | 8/9 **agreed** (88.9%) | – | | | | | |
| The threshold for comprehensive disease control should be no rectal bleeding, PRO2/pMCS rectal bleeding domain score 0. | 9/9 **agreed** (100%) | – | | | | | |
| **Urgency** | | | | | | | |
| Urgency is important to consider when assessing comprehensive disease control. | 9/9 **agreed** (100%) | – | | | | | |
| Urgency should be included in a measure of comprehensive disease control. | 9/9 **agreed** (100%) | – | | | | | |
| A simple system using natural language can be used when assessing the severity and frequency of urgency (e.g. none, mild, moderate or severe) | 6/9 **agreed** (66.7%) | [Alternative scoring system met consensus and so statement not modified and re-asked] | | | | | |
| A numerical scale or scoring system (e.g. using the Urgency NRS measurement tool) can be used when assessing the severity and frequency of urgency. | 7/9 **agreed** (77.8%) | – | | | | | |
| The threshold for comprehensive disease control should be mild urgency occasionally  (< 2 days/week). | 6/9 **agreed** (66.7%) | Urgency should be absent for remission to be considered comprehensive | 7/9 **agreed** (77.8%) | Urgency should be absent for remission to be considered comprehensive, with the exception of mild urgency, if patients do not find this impactful. | | 9/9 **agreed** (100%) | |
|  |  | Some urgency can occur and remission can still be considered to be comprehensive | 5/9 **agreed** (44.4%) |  |  |  |  |
| **Stool frequency/diarrhoea** | | | | | | | |
| Stool frequency/diarrhoea is important to consider when assessing comprehensive disease control. | 9/9 **agreed** (100%) | – | | | | | |
| Assessment of stool frequency/diarrhoea should be included in a measure of comprehensive disease control. | 9/9 **agreed** (100%) | – | | | | | |
| Stool frequency/diarrhoea can be assessed from counting the number of stools per day. | 9/9 **agreed** (100%) | – | | | | | |
| Stool frequency/diarrhoea can be assessed from counting the number of stools per day and using the thresholds in PRO2/pMCS. | 8/9 **agreed** (88.9%) | – | | | | | |
| The threshold signifying remission should be 1–2 stools per day more than is normal for them or PRO2/pMCS stool frequency domain score 1. | 7/9 **agreed** (77.8%) | – | | | | | |
| **Abdominal pain** | | | | | | | |
| Abdominal pain is important to consider when assessing comprehensive disease control. | 8/9 **agreed** (88.9%) | – | | | | | |
| Abdominal pain should be included in a measure of comprehensive disease control. | 7/9 **agreed** (77.8%) | – | | | | | |
| A simple system can be used when assessing the severity and frequency of abdominal pain (e.g. none, mild, moderate or severe; 0 to 10). | 9/9 **agreed** (100%) | – | | | | | |
| A numerical scale (e.g. using the VAS) can be used when assessing the severity and frequency of abdominal pain. | 8/9 **agreed** (88.9%) | – | | | | | |
| The threshold for comprehensive disease control should be mild pain occasionally  (< 2 days/week). | 6/9 **agreed** (66.7%) | Abdominal pain should be absent for remission to be considered comprehensive | 7/9 **agreed** (77.8%) | Abdominal pain should be absent for remission to be considered comprehensive, with the exception of mild abdominal pain, if patients do not find this impactful. | | 9/9 **agreed** (100%) | |
|  |  | Some abdominal pain can occur and remission can still be considered to be comprehensive | 6/9 **agreed** (66.7%) |  |  |  |  |
| **Disease-related QoL** | | | | | | | |
| Disease-related QoL is important to consider when assessing comprehensive disease control. | 9/9 **agreed** (100%) | – | | | | | |
| Disease-related QoL should be included in a measure of comprehensive disease control. | 9/9 **agreed** (100%) | – | | | | | |
| The SIBDQ or IBD-disk should be used to measure disease-related QoL. | 7/9 **agreed** (77.8%) | – | | | | | |
| The threshold for comprehensive disease control for disease-related QoL should be no disability, or IBD-disk ≤ 24. | 7/9 **agreed** (77.8%) | – | | | | | |
| – | | Disease-related QoL (e.g. assessed using the SIBDQ or IBD-Disk) is more important than health-related QoL (e.g. assessed using the EQ-5D or SF-36) | 8/9 agreed (88.9%) | – | | | |
| **Extraintestinal manifestations** | | | | | | | |
| Extraintestinal manifestations are important to consider when assessing comprehensive disease control. | 9/9 **agreed** (100%) | – | | | | | |
| Extraintestinal manifestations have the same importance as non-extraintestinal manifestations, yes or no? | 6/9 voted **yes** (66.7%) | [Consensus not reached so aspect not considered further] | | | | | |
| Extraintestinal manifestations should be included in a measure of comprehensive disease control. | 8/9 **agreed** (88.9%) | – | | | | | |
| **Fatigue** | | | | | | | |
| Fatigue is important to consider when assessing comprehensive disease control. | 7/9 **agreed** (77.8%) | – | | | | | |
| Fatigue should be included in a measure of comprehensive disease control. | 8/9 **agreed** (88.9%) | – | | | | | |
| A simple system can be used when assessing the severity and frequency of fatigue (e.g. none, mild, moderate or severe; 0 to 10). | 8/9 **agreed** (88.9%) | – | | | | | |
| A numerical scale/scoring system (e.g. the IBD-F/IBD-F SCORE1 component [evaluated level and duration of fatigue] or FACIT-F) can be used when assessing the severity and frequency of fatigue. | 7/9 **agreed** (77.8%) | – | | | | | |
| The threshold for comprehensive disease control should be mild fatigue occasionally  (< 2 days/week). | 6/9 **agreed** (66.7%) | Fatigue should be absent for remission to be considered comprehensive | 6/9 **agreed** (66.7%) | For patients who experienced impactful fatigue when their UC was active, comprehensive disease control should be characterized by a meaningful reduction in fatigue, excluding any fatigue resulting from other obvious non-UC-related causes | | 9/9 **agreed** (100%) | |
|  |  | Some fatigue can occur and remission can still be considered to be comprehensive | 8/9 **agreed** (88.9%) |  |  |  |  |
| **Sleep disturbance** | | | | | | | |
| Sleep disturbance is important to consider when assessing comprehensive disease control. | 6/9 **agreed** (66.7%) | Sleep disturbance is important to consider when assessing comprehensive disease control. | 7/9 **agreed** (77.8%) | – | | | |
| Sleep disturbance should be included in a measure of comprehensive disease control. | 7/9 **agreed** (77.8%) | Sleep disturbance should be included in a measure of comprehensive disease control. | 6/9 **agreed** (66.7%) | Sleep disturbance should be included in a measure of comprehensive disease control. | | 9/9 **agreed** (100%) | |
| A simple system can be used when assessing the severity and frequency of sleep disturbance (e.g. none, mild, moderate or severe; 0 to 10). | 6/9 **agreed** (66.7%) | If evaluating sleep disturbance, a simple system can be used when assessing the severity and frequency of sleep disturbance (e.g. none, mild, moderate or severe; 0 to 10). | 6/9 **agreed** (66.7%) | – | | | |
| A numerical scale/scoring system can be used when assessing the severity and frequency of sleep disturbance. | 6/9 **agreed** (66.7%) | If evaluating sleep disturbance, a numerical scale/scoring system (e.g. using an instrument like the PROMIS Sleep Disturbance Item Bank^2^) can be used. | 9/9 **agreed** (100%) | – | | | |
| The threshold for comprehensive disease control should be mild sleep disturbance occasionally (< 2 days/week). | 5/9 **agreed** (55.6%) | There is not enough evidence for a threshold for sleep disturbance to assess comprehensive disease control. | 9/9 **agreed** (100%) | For patients who experienced impactful sleep disturbance when their UC was active, comprehensive disease control should be characterized by a meaningful reduction in sleep disturbance, excluding any sleep disturbance resulting from other obvious non-UC-related causes | | 9/9 **agreed** (100%) | |
| ***Objective measures of disease activity*** | | | | | | | |
| **Endoscopic remission** | | | | | | | |
| Endoscopic remission is important to consider when assessing comprehensive disease control | 9/9 **agreed** (100%) | – | | | | | |
| Endoscopic remission should be included in a measure of comprehensive disease control | 9/9 **agreed** (100%) | – | | | | | |
| The MES or UCEIS should be used to assess endoscopic remission, using the threshold ≤ 1 | 9/9 **agreed** (100%) | – | | | | | |
| Is there enough evidence to suggest the best timing of endoscopy and evaluate whether response-guided assessments are more appropriate than performing endoscopies at fixed intervals? | 6/8^a^ voted **No** (75%) | – | | | | | |
| **Inflammatory biomarkers** | | | | | | | |
| Inflammatory biomarkers provide a non-invasive measure that can be used to monitor patients and should be performed regularly to allow early detection of disease activity. | 9/9 **agreed** (100%) | – | | | | | |
| Inflammatory biomarkers should be included in a measure of comprehensive disease control. | 9/9 **agreed** (100%) | – | | | | | |
| The threshold for remission for FC should be ≤ 100–250 μg/g and the threshold for CRP should be below the upper limit of normal. | 8/9 **agreed** (88.9%) | – | | | | | |
| FC and CRP levels should be given equal weighting when assessing remission. | 8/9 **disagreed** (88.9%) | – | | | | | |
| FC levels should be prioritized over CRP levels when assessing remission. | 9/9 **agreed** (100%) | – | | | | | |
| CRP levels should be prioritized over FC levels when assessing remission. | 7/9 **disagreed** (77.8%) | – | | | | | |
| **Histology** | | | | | | | |
| Histology provides information on inflammatory disease activity. | 9/9 **agreed** (100%) | – | | | | | |
| Histology should be included in a measure of comprehensive disease control. | 6/9 **agreed** (66.7%) | Rank the following statements:  1. Histology should not be included in a measure of comprehensive disease control  2. Histological response/improvement should be included in a measure of comprehensive disease control  3. Histological remission should be included in a measure of comprehensive disease control | 1. 55.6% first choice  2. 33.3 first choice  3. 0% first choice | Histological inflammatory activity should be absent for remission to be considered comprehensive | | 9/9 **agreed** (100%) | |
| The threshold for histological remission should be a non-inflammatory state | 9/9 **agreed** (100%) | [Superseded in voting round 3] | | |  | |  |
| The threshold for histological remission should be complete resolution of changes | 8/9 **agreed** (88.9%) | [Superseded in voting round 3] | | |  |  |  |
| **Ultrasound** | | | | | | | |
| Ultrasound can assess mucosal healing and are important to consider when assessing comprehensive disease control. | 7/9 **agreed** (77.8%) | – | | | Ultrasound and other imaging techniques can assess mucosal healing and are important to consider when assessing comprehensive disease control. | | – |
| Ultrasound should be included in a measure of comprehensive disease control. | 4/9 **agreed** (44.4%) | Ultrasound should not be included in a measure of comprehensive disease control | 5/9 **agreed** (55.6%) | [Discarded as did not meet consensus] | | | |
| Ultrasound results should be assessed using the UC-IUS index. | 7/9 **agreed** (77.8%) | – | | | | | |
| The threshold for remission using ultrasound should be bowel wall thickness ≤ 3.2 mm. | 7/9 **agreed** (77.8%) | – | | | | | |
| ***Combination therapies*** | | | | | | | |
| Discontinuation of corticosteroids is important to consider when assessing comprehensive disease control. | 9/9 **agreed** (100%) | – | | | | | |
| Discontinuation of azathioprine is important to consider when assessing comprehensive disease control. | 7/9 **disagreed** (77.8%) | – | | | | | |
| Discontinuation of 5-ASA is important to consider when assessing comprehensive disease control. | 7/9 **disagreed** (77.8%) | – | | | | | |
| Discontinuation of corticosteroids should be included in a measure of comprehensive disease control. | 9/9 **agreed** (100%) | – | | | | | |
| Discontinuation of azathioprine should be included in a measure of comprehensive disease control. | 7/9 **disagreed** (77.8%) | – | | | | | |
| Discontinuation of 5-ASA should be included in a measure of comprehensive disease control. | 7/9 **disagreed** (77.8%) | – | | | | | |
| – | | Discontinuation of biologics or small molecules is important to consider when assessing comprehensive disease control. | 9/9 **disagreed** (100%) | – | | | |
| – | | Discontinuation of biologics or small molecules should be included in a measure of comprehensive disease control. | 8/9 **disagreed** (88.9%) | – | | | |

^a^One panel member did not answer this question because it was beyond their professional knowledge.

5-ASA, 5-aminosalicylic acid; CRP, C-reactive protein; EQ-5D, EuroQoL-5 dimensions; FACIT-F, Functional Assessment of Chronic Illness Therapy – Fatigue; FC, faecal calprotectin; IBD-Disk; inflammatory bowel disease-disk; IBD-F, inflammatory bowel disease-fatigue; MES, Mayo endoscopic score; NRS, numeric rating scale; pMCS, partial Mayo Clinic Score; PRO2, 2-item patient-reported outcome; PROMIS, Patient-Reported Outcomes Measurement Information System; QoL, quality of life; SF-36, short-form-36; SIBDQ, short inflammatory bowel disease questionnaire; UC, ulcerative colitis; UCEIS, Ulcerative Colitis Endoscopic Index of Severity; UC-IUS, ulcerative colitis-intestinal ultrasound; VAS, visual analogue scale.

Supplementary Table 8. Overview of commonly used thresholds for inflammatory biomarkers.

|  | SLR findings (most common underlined) | Physician survey results |
| --- | --- | --- |
| FCP | 0.5 mg/dL, 50 mg/kg, 100 μg/g, 150 μg/g, 223 mg/L, 250 μg/g, 340 μg/g^3-28^ | 100 to 250 μg/g |
| CRP | 5 mg/L, 5.5 mg/L^3,4,8,11,12,21,24,27,29-38^ | > ULN to 5 mg/L |

CRP, C-reactive protein; FCP, faecal calprotectin; SLR, systematic literature review; ULN, upper limit of normal.

# References

1. NIHR. PROSPERO. International propsective register of systematic reviews. Available at: <https://www.crd.york.ac.uk/prospero/> (accessed 16 December 2022).

2. Buysse DJ, Yu L, Moul DE*, et al.* Development and validation of patient-reported outcome measures for sleep disturbance and sleep-related impairments. *Sleep* 2010;**33**:781–92.

3. Golovics PA, Gonczi L, Reinglas J*, et al.* Patient-reported outcome and clinical scores are equally accurate in predicting mucosal healing in ulcerative colitis: A prospective study. *Dig Dis Sci* 2022;**67**:3089–95.

4. Park J, Kang SJ, Yoon H*, et al.* Histologic evaluation using the robarts histopathology index in patients with ulcerative colitis in deep remission and the association of histologic remission with risk of relapse. *Inflamm Bowel Dis* 2022;**28**:1709–16.

5. Nigam GB, Limdi JK, Hamdy S, Vasant DH. PTH-108 The hidden burden of faecal incontinence in active and quiescent ulcerative colitis: an underestimated problem? *Gut* 2019;**68**:A87–A.

6. Bertani L, Blandizzi C, Mumolo MG*, et al.* Fecal calprotectin predicts mucosal healing in patients with ulcerative colitis treated with biological therapies: A prospective study. *Clin Transl Gastroenterol* 2020;**11**:e00174.

7. Cannatelli R, Bazarova A, Zardo D*, et al.* Fecal calprotectin thresholds to predict endoscopic remission using advanced optical enhancement techniques and histological remission in IBD patients. *Inflamm Bowel Dis* 2021;**27**:647–54.

8. Grimstad T, Norheim KB, Isaksen K*, et al.* Fatigue in newly diagnosed inflammatory bowel disease. *J Crohns Colitis* 2015;**9**:725–30.

9. Naganuma M, Hirai F, Kobayashi K*, et al.* Middle-term prognosis in patients with ulcerative colitis who achieved clinical and endoscopic remission by budesonide rectal foam. *PLoS One* 2019;**14**:e0220413.

10. Macedo Silva V, Lima Capela T, Freitas M*, et al.* P0322 platelet-to-lymphocyte ratio index: Still not above fecal calprotectin for non-invasive assessment of endoscopic activity in ulcerative colitis. Presented at the United European Gastroenterology congress, 3–5 October 2021 (Virtual). In: United European Gastroenterology (UEG).

11. Matsubayashi M, Kobayashi T, Okabayashi S*, et al.* Determining the usefulness of Capsule Scoring of Ulcerative Colitis in predicting relapse of inactive ulcerative colitis. *J Gastroenterol Hepatol* 2021;**36**:943–50.

12. Pop CS, Filip PV, Diaconu SL, Matei C, Furtunescu F. Correlation of biomarkers with endoscopic score: Ulcerative colitis endoscopic index of severity (UCEIS) in patients with ulcerative colitis in remission. *Medicina (Kaunas)* 2020;**56**:45.

13. Hart L, Chavannes M, Kherad O*, et al.* Faecal calprotectin predicts endoscopic and histological activity in clinically quiescent ulcerative colitis. *J Crohns Colitis* 2020;**14**:46–52.

14. João M, Dominguez F, Portela F, Narra Figueiredo P. Pain in ulcerative colitis: A forgotten patient-reported outcome In: United European Gastroenterology (UEG), 2021.

15. Kawashima K, Oshima N, Yuki T*, et al.* Sa1876 low fecal calprotectin level predicts histological healing and prolonged clinical remission in ulcerative colitis patients with clinical remission and mucosal healing. *Gastroenterol* 2020;**158**:S–461–S–2.

16. Kim ES, Lee HS, Kim SK*, et al.* Fecal calprotectin is more accurate than fecal immunochemical test for predicting mucosal healing in quiescent ulcerative colitis: a prospective multicenter study. *Scand J Gastroenterol* 2020;**55**:163–8.

17. Lindholm M, Godskesen LE, Manon-Jensen T*, et al.* Endotrophin and C6Ma3, serological biomarkers of type VI collagen remodelling, reflect endoscopic and clinical disease activity in IBD. *Sci Rep* 2021;**11**:14713.

18. Magro F, Lopes J, Borralho P*, et al.* Comparison of the Nancy Index With Continuous Geboes Score: Histological Remission and Response in Ulcerative Colitis. *J Crohns Colitis* 2020;**14**:1021–5.

19. Magro F, Lopes J, Borralho P*, et al.* Comparing the continuous geboes score with the robarts histopathology index: Definitions of histological remission and response and their relation to faecal calprotectin levels. *J Crohns Colitis* 2020;**14**:169–75.

20. Malvao LDR, Madi K, Esberard BC*, et al.* Fecal calprotectin as a noninvasive test to predict deep remission in patients with ulcerative colitis. *Medicine (Baltimore)* 2021;**100**:e24058.

21. Mavropoulou E, Mechie NC, Knoop R*, et al.* Association of serum interleukin-6 and soluble interleukin-2-receptor levels with disease activity status in patients with inflammatory bowel disease: A prospective observational study. *PLoS One* 2020;**15**:e0233811.

22. Mechie NC, Mavropoulou E, Ellenrieder V*, et al.* Serum vitamin D but not zinc levels are associated with different disease activity status in patients with inflammatory bowel disease. *Medicine (Baltimore)* 2019;**98**:e15172.

23. Nakov RV, Nakov VN, Gerova VA, Tankova LT. Role of fecal calprotectin as a noninvasive indicator for ulcerative colitis disease activity. *Folia Med (Plovdiv)* 2019;**61**:188–96.

24. Pinto-Lopes P, Afonso J, Pinto-Lopes R*, et al.* Serum Dipeptidyl Peptidase 4: A Predictor of Disease Activity and Prognosis in Inflammatory Bowel Disease. *Inflamm Bowel Dis* 2020;**26**:1707–19.

25. Sakuraba A, Nemoto N, Hibi N*, et al.* Extent of disease affects the usefulness of fecal biomarkers in ulcerative colitis. *BMC Gastroenterol* 2021;**21**:197.

26. Sakurai T, Yoshihiro A, Haruna M*, et al.* P278 Comparison of prostaglandin E–major urinary metabolite (PGE-MUM) with faecal calprotectin and faecal immunochemical tests for determining endoscopic remission in patients with ulcerative colitis. *J Crohns Colitis* 2020;**14**:S290–S.

27. Shinzaki S, Matsuoka K, Tanaka H*, et al.* Leucine-rich alpha-2 glycoprotein is a potential biomarker to monitor disease activity in inflammatory bowel disease receiving adalimumab: PLANET study. *J Gastroenterol* 2021;**56**:560–9.

28. Yen HH, Chen MW, Chang YY*, et al.* Predictive values of stool-based tests for mucosal healing among Taiwanese patients with ulcerative colitis: a retrospective cohort analysis. *PeerJ* 2020;**8**:e9537.

29. Cifci S, Ekmen N. Prediction of mucosal health by NLR, CRP x NLR and MPV in ulcerative colitis: Can their availability change according to treatment options? *Cureus* 2021;**13**:e19942.

30. Wakai M, Hayashi R, Tanaka S*, et al.* Serum amyloid A is a better predictive biomarker of mucosal healing than C-reactive protein in ulcerative colitis in clinical remission. *BMC Gastroenterol* 2020;**20**:85.

31. Bakkaloglu OK, Eskazan T, Celik S*, et al.* Can we predict mucosal remission in ulcerative colitis more precisely with a redefined cutoff level of C-reactive protein? *Colorectal Dis* 2022;**24**:77–84.

32. Chen YH, Wang L, Feng SY*, et al.* The relationship between c-reactive protein/albumin ratio and disease activity in patients with inflammatory bowel disease. *Gastroenterol Res Pract* 2020;**2020**:3467419.

33. Coskun Y. Role of mean platelet volume as a marker of disease activity in patients with ulcerative colitis. *Acta Medica Mediterranea* 2019;**35(6)**:3355–60.

34. Endo K, Satoh T, Yoshino Y*, et al.* Neutrophil-to-lymphocyte and platelet-to-lymphocyte ratios as noninvasive predictors of the therapeutic outcomes of systemic corticosteroid therapy in ulcerative colitis. *Inflamm Intest Dis* 2021;**6**:218–24.

35. Shin J, Kong SM, Seong G, Kim YH. What is the appropriate cut-off value of CRP to predict endoscopic remission in patients with ulcerative colitis in clinical remission? *Int J Colorectal Dis* 2020;**35**:2249–55.

36. Liu A, Lv H, Tan B*, et al.* Accuracy of the highly sensitive C-reactive protein/albumin ratio to determine disease activity in inflammatory bowel disease. *Medicine (Baltimore)* 2021;**100**:e25200.

37. Mak JWY, Yuen NTK, Yip TCF*, et al.* P296 Stopping 5-aminosalicylic acid in patients with ulcerative colitis who are in clinical remission does not increase risks of flare. *J Crohns Colitis* 2021;**15**:S325–S6.

38. Sharma D, Choden T, Halaseh R*, et al.* Sa078 clinical and laboratory markers do not predict deep histologic remission in patients with ulcerative colitis. *Gastroenterol* 2021;**160**:S–411–S–2.
