# Supplementary material for: Defining Comprehensive Disease Control for Use as a Treatment Target for Ulcerative Colitis in Clinical Practice: International Delphi Consensus Recommendations
Source: J Crohns Colitis. 2023 Aug 16;18(1):91–105. doi: 10.1093/ecco-jcc/jjad130 (PMC10821705; doi:10.1093/ecco-jcc/jjad130)
Supplement: jjad130_suppl_Supplementary_Materials [file jjad130_suppl_supplementary_materials.docx]

**Plain language summary**

**Defining Comprehensive Disease Control for use as a Treatment Target for Ulcerative Colitis in Clinical Practice: International Delphi Consensus Recommendations**

Stefan Schreiber, Silvio. Danese, Axel Dignass, Eugeni Domènech, Massimo C. Fantini, Marc Ferrante, Jonas Halfvarson, Ailsa Hart, Fernando Magro, Charlie W. Lees, Salvo Leone, Marieke J. Pierik, Michele Peters, Polly Field, Helen Schofield, Laurent Peyrin-Biroulet.

Ulcerative colitis (UC) is an inflammatory disease of the gut lining. Patients with UC may have long periods of time without the signs and symptoms of disease, known as remission. When doctors assess if patients are in remission, they mainly look at specific symptoms, such as how often they go to the toilet and if there is blood in their stool. This is because this is how they have been assessing UC for decades and it is how the effectiveness of new treatments is assessed in studies. However, patients in remission may still have other symptoms that impact their life, and these other symptoms may affect their life more than going to the toilet regularly or having blood in their stool. As well, patients want to feel like their disease does not affect them and they want to look at all their symptoms together, instead of looking at each symptom separately.

We want to create a new definition of remission that that is ‘comprehensive’, meaning it includes all aspects of UC that affects patients on an individual level. We are calling this definition ‘comprehensive disease control’. To do this, we asked 18 patients about their symptoms when they are in remission and asked ten doctors about their experience treating patients. We also looked at research already published by other scientists. We then asked ten expert doctors and one patient expert to vote in three voting rounds on whether they agree on including various aspects in comprehensive disease control.

Overall, the experts agreed that our definition of comprehensive disease control should include certain symptoms, the results of certain tests that doctors use to see if the gut is inflamed, and if patients are taking a type of drug that suppresses the immune system called corticosteroids. The symptoms in the definition were blood in the stool, how often patients go to the toilet, their perceived physical and mental health over time, how urgent their need is for the toilet, stomach pain, fatigue, problems sleeping, and other symptoms not related to the gut. The tests in the definition were 1) endoscopy, where a flexible camera is inserted into the rectum; 2) measuring levels of biological markers of inflammation in the stool or blood, and 3) histology, where a doctors take a small sample of the gut to look at tissues and cells under a microscope. We recommend that doctors talk to patients about these symptoms (or use questionnaires) and use these tests. All this information will help doctors and patients choose the treatment to help each individual patient achieve comprehensive disease control.

**Word count:** 429
